# Sustainable Valorization of Oil Palm Coproducts: Physicochemical Characterization and Potential Use in Insect Bioconversion

**DOI:** 10.3390/foods15101754

**Published:** 2026-05-15

**Authors:** Fabiane Cerqueira de Almeida, Débora Pereira Rodrigues Borges, Lorena Lindsey Coelho Duarte Santos, Jade Silva Oliveira, Cláudio Vaz Di Mambro Ribeiro, Luís Fernandes Pereira Santos, Camila Duarte Ferreira Ribeiro, Lucas Guimarães Cardoso, Denilson de Jesus Assis, Jania Betânia Alves da Silva, Renata Quartieri Nascimento, Ederlan de Souza Ferreira, Kodjovi Ayena, Marcelo Andres Umsza-Guez, Carolina Oliveira de Souza

**Affiliations:** 1 Graduate Program in Food Science, College of Pharmacy, Federal University of Bahia, Rua Barão de Jeremoabo, 147, Ondina, Salvador 40170-115, BA, Brazil; deboraprborges@gmail.com (D.P.R.B.); claudioribeiro@ufba.br (C.V.D.M.R.); ederlan.ferreira@ufba.br (E.d.S.F.); kayena@ufba.br (K.A.); marcelo.umsza@ufba.br (M.A.U.-G.); 2 Graduate Program in Food, Nutrition and Health, Federal University of Bahia, Basílio da Gama Street, Rua Basilio da Gama-w/n-Campus Canela, Salvador 40110-907, BA, Brazil; lorenalindsey203@gmail.com (L.L.C.D.S.); luisfernandes@ufba.br (L.F.P.S.); camiladuartef@ufba.br (C.D.F.R.); 3 Department of Animal Science, School of Veterinary Medicine and Animal Science, Federal University of Bahia, Salvador 40170-110, BA, Brazil; 21jadeso@gmail.com; 4 Graduate Program in Chemical Engineering, Polytechnic School, Federal University of Bahia, Salvador 40210-630, BA, Brazil; guimaraes.lucas@animaeducacao.com.br (L.G.C.); janiabetania@ufrb.edu.br (J.B.A.d.S.); 5 School of Exact and Technological Sciences, Salvador University, Salvador 41820-020, BA, Brazil; denilson.assis@animaeducacao.com.br; 6 Center for Exact and Technological Sciences, Faculty of Mechanical Engineering, Federal University of Recôncavo da Bahia, Cruz das Almas 44380-000, BA, Brazil; 7 Department of Life Sciences, State University of Bahia, Salvador 40411-120, BA, Brazil; rqnutri@gmail.com; 8 College of Pharmacy, Federal University of Bahia, Salvador 40170-115, BA, Brazil

**Keywords:** edible insects, PKC, mesocarp fiber, carotenoids, circular economy

## Abstract

The oil palm production chain generates coproducts whose sustainable valorization remains a challenge. This study tested the hypothesis that partial replacement of the conventional substrate with oil palm coproducts could maintain the productive performance of *Zophobas atratus* larvae and generate value-added biomass. Mesocarp fiber (MF), palm oil mill effluent (POME), and palm kernel cake (PKC) were characterized in terms of physicochemical composition, carotenoids, and antioxidant capacity and examined as partial substitutes for wheat bran in six diets for *Z. atratus*. PKC demonstrated higher levels of protein (15.27%), carbohydrates (65.68%), neutral detergent fiber (68.35%), acid detergent fiber (37.70%), and saturated fatty acids (83.06%) and greater antioxidant capacity associated with phenolic compounds. MF showed the highest carotenoid content (138.27 mg/100 g), and POME had the highest lipid content (17.69%). Diet containing 50% PKC-supplemented wheat bran promoted higher feed conversion efficiency (78.99%), lower feed conversion ratio (0.90%), and higher larval protein content (39.14%) and maintained performance similar to that of the control. Larvae fed on 50% MF exhibited carotenoid bioaccumulation, with >190% increase compared with the control. Although the coproducts demonstrate potential as substrates, mortality restricts their technical feasibility. Their use depends on an adequate protein/energy balance and the digestibility of the fibrous fraction for strategic supplementation.

## 1. Introduction

Palm oil (*Elaeis guineensis*), is the most widely produced and traded vegetable oil worldwide, with applications in the food, cosmetic, pharmaceutical, and biofuel sectors, especially for biodiesel production. Palm oil refers to the refined product used industrially, whereas dendê oil corresponds to the crude, non-refined form traditionally consumed in Brazil [1,2].

In 2024, global vegetable oil production reached 228.70 million tons, with palm oil accounting for 34.52% of this total, despite occupying only 5.5% of the land allocated for vegetable oil production [1,3]. Indonesia and Malaysia together account for ~85% of global palm oil production [3,4].

Although palm oil production exhibits high productive efficiency, the expansion of the oil palm supply chain is associated with significant environmental effects, including deforestation, biodiversity loss, greenhouse gas emissions, and the generation of waste and coproducts [5,6]. During industrial processing, only approximately 10% of the fruit is converted into oil, whereas ~90% corresponds to residues and coproducts [6]. Among the major coproducts are empty fruit bunches (22%), mesocarp fiber (12%), palm kernel shells (5%), palm kernel cake (3.5%), and palm oil mill effluent, which may represent 50–60% of the volume generated during processing [4,7,8]. In particular, the inadequate disposal of these materials, often through burning or discharge into water bodies, intensifies environmental degradation [4].

Among the abovementioned coproducts, mesocarp fiber is a bulky, lignocellulosic, and fibrous biomass obtained after pressing the mesocarp of oil palm fruits [9]. Palm oil mill effluent, abbreviated as (POME) and popularly known as *bambá* [8], corresponds to the aqueous fraction generated during the extraction and purification of palm oil and consists of water, oil, and suspended solids, in addition to carotenoids, lignin, pectin, and tannins [10]. Palm kernel cake is derived from the extraction of oil from the seed endosperm and is characterized by a high fiber content and moderate levels of protein and fat [11].

Although several valorization strategies have been proposed, such as the use of empty fruit bunches for energy [8], the application of mesocarp fiber as fertilizer [12], the utilization of palm kernel cake in animal feeding [1,13], and the use of POME for soap production [8,14], these approaches present limitations in terms of value addition, economic stability, and systemic integration. Therefore, there exists a need to explore biotechnological pathways capable of converting these coproducts into ingredients with higher strategic value, such as their application within the alternative protein supply chain.

In this context, biorefineries based on edible insects are emerging as promising platforms for the bioconversion of agro-industrial residues. These organisms demonstrate high feed conversion efficiency and the ability to transform low-value organic substrates into biomass rich in proteins, lipids, and chitin, thereby contributing to production systems aligned with the principles of circular economy and sustainable bioeconomy [15,16,17]. Furthermore, the production of insects as an alternative protein source may help reduce dependence on conventional ingredients, such as soybean meal and fishmeal, thereby mitigating the environmental pressures associated with these production chains [18].

Among species with biotechnological potential, *Zophobas atratus* excels due to its high growth rate and feed conversion efficiency [16,19,20]. Nevertheless, although several agro-industrial byproducts have been investigated as substrates for insects, such as brewer’s spent grain [21], grape pomace [19,22], olive pomace [23], and flaxseed cake [16], coproducts from the oil palm production chain have been scarcely explored as substrates for bioconversion.

Based on the above-described information, it is hypothesized that coproducts from the oil palm production chain, when used as feed substrates, can promote the growth of *Z. atratus*, resulting in zootechnical performance comparable to that obtained with a conventional diet and in biomass with a nutritional composition suitable for application in animal feeding.

The novelty of this study lies in the integrated evaluation of coproducts from the oil palm production chain as substrates for *Z. atratus*, combining the physicochemical characterization of the residues, diet formulation, and analysis of the productive performance and nutritional composition of the resulting biomass. To our knowledge, no studies have yet explored this approach to this species.

Therefore, the objectives of this study were to characterize, in terms of physicochemical characteristics, fatty acid profile, total carotenoids, total phenolics, and antioxidant activity, the byproducts of the oil palm production chain and to investigate their potential use by *Z. atratus* for bioconversion.

## 2. Materials and Methods

### 2.1. Coproducts

Palm oil mill effluent (POME) and mesocarp fiber (MF) were donated by Dandouro (Valença, BA, Brazil), and palm kernel cake (PKC) was obtained from Óleos de Palma S.A Agroindustrial (Taperoá, BA, Brazil). For physicochemical analyses, the samples were used in their whole form; mesocarp fiber and palm kernel cake were ground using a grain grinder (Cadence, MDR302-150 W, São Paulo-SP, Brazil), and the POME was homogenized to ensure sample representativeness. Then, all samples were stored frozen until further analysis. Wheat bran (WB) (Grão, Salvador, Bahia, Brazil) was purchased from a local retailer and consisted of 10.10% moisture, 5.09% ash, 8.07% protein, 4.06% fat, 72.68% carbohydrates, and 10.8 mg/100 g carotenoids. Wheat bran is widely used as a diet for insects because it balances nutritional requirements and is inexpensive [24]. *Z. atratus* larvae were obtained from Biofábrica São Luís (São Luís, MA, Brazil).

### 2.2. Physicochemical Characterization

The physicochemical composition of the coproducts was determined according to the methodology of the Association of Official Analytical Chemists [25]. Moisture content was determined by oven drying (DeLeo TLK48, Porto Alegre-RS, Brazil) at 105 °C until constant mass (AOAC 925.09), and ash content was determined in a muffle furnace (Lavoisier 402-D, São Paulo, Brazil) by incineration at 550 °C (AOAC 942.05). Crude protein content was determined using the Kjeldahl method (AOAC 991.20) with a conversion factor of 6.25 for the coproducts. The Soxhlet method was used for determining total lipids (AOAC 920.39). Carbohydrate content was calculated using difference [26], and the energy value was determined considering 4 kcal/g for carbohydrates and proteins and 9 kcal/g for lipids [27]. Neutral detergent fiber (NDF) and acid detergent fiber (ADF) were determined according to the methodology described by Van Soest, Robertson, and Lewis [28].

### 2.3. Fatty Acid Profile

Fatty acid methyl esters (FAME) were obtained by derivatization of the fatty acids present in the total lipids previously extracted using a cold method [29], as described by Nascimento et al. [19]. For this purpose, an aliquot of total lipids (25 mg) was subjected to alkaline methylation using a methanolic NaOH solution (0.5 M), followed by treatment with a methanolic BF_3_ solution (12%, m v^−1^) and the subsequent extraction of FAME using isooctane.

FAME were separated using a gas chromatograph (PerkinElmer Clarus 680, Waltham, MA, USA) equipped with a flame ionization detector and a DB–Fast FAME column (30 m × 0.25 mm × 0.25 μm). The analytical conditions were as follows: injector temperature of 250 °C, detector temperature of 280 °C, and an oven temperature program starting at 60 °C for 0.5 min, increasing at 25 °C/min to 194 °C (held for 1 min), followed by an increase of 5 °C/min to 235 °C and maintaining for 1 min. Helium was used as the carrier gas at a flow rate of 1.0 mL/min, and 1-μL injections were performed in split mode (1:50).

Fatty acids were identified by comparing the retention times of sample peaks with those of fatty acid methyl esters from a standard mixture (C4–C24, 189-19, Sigma-Aldrich, St. Louis, MO, USA), and their relative percentages were determined by normalization of the peak area. The sums of the levels of saturated fatty acids (SFA), monounsaturated fatty acids (MUFA), and polyunsaturated fatty acids (PUFA) were calculated based on the fatty acid profile.

### 2.4. Total Carotenoids

The total carotenoids were extracted according to a methodology adapted from Rodriguez-Amaya [30], as studies indicate that the saponification step may be omitted due to degradation and loss of total or individual carotenoids [31,32]. Therefore, a 1 g sample was macerated with acetone until complete pigment extraction, followed by vacuum filtration. The extract was transferred to a separatory funnel containing distilled water and petroleum ether, followed by the addition of anhydrous sodium sulfate as a drying agent. The organic phase containing the carotenoids was collected and brought to volume in a 25 mL volumetric flask containing petroleum ether. Quantification was performed using a spectrophotometer (PerkinElmer Lambda 35, Waltham, MA, USA) at 445 nm using a β-carotene standard curve (Sigma-Aldrich, USA) (y = 0.0135x + 0.1514; R^2^ = 0.9956).

### 2.5. Total Phenolics

For determining the total phenolic compounds, the extract was obtained according to a methodology adapted from Castro [33] using 1 g of sample diluted in 10 mL of 70% ethanol under agitation in a magnetic stirrer (Tecnal, TE-0851, Piracicaba-SP, Brazil) for 30 min. The sample was then centrifuged (Eppendorf Centrifuge 5702 R, Hamburg, Germany) for 10 min at 4000 rpm at a temperature of 25 °C. The resulting supernatant was filtered and transferred to a 25 mL volumetric flask, and the volume was adjusted using the extraction solvent. The quantification of phenolic compounds was achieved according to the method described by Singleton and Rossi [34] using gallic acid as the standard. Briefly, 0.1 mL of extract, 0.5 mL of Folin reagent (0.2 M), 1.5 mL of sodium carbonate (20%), and 7.9 mL of distilled water were added to a test tube. After 1 h, the samples were measured using a spectrophotometer (PerkinElmer Lambda 35, Waltham, MA, USA) at 765 nm, and the results were expressed as mg GAE/g (calibration curve: y = 0.0019x + 0.0024; R^2^ = 0.9955).

### 2.6. Antioxidant Activity

The DPPH (2,2-diphenyl-1-picrylhydrazyl) radical scavenging activity was determined according to the methodology described by Rufino et al. [35], with the absorbance measured using a spectrophotometer (PerkinElmer Lambda 35, Waltham, MA, USA) at 515 nm. Trolox (238813, Sigma-Aldrich, St. Louis, MO, USA) was used as the standard, and the results were expressed as g sample/g DPPH (calibration curve: y = 0.0146x + 0.0077; R^2^ = 0.9991). The antioxidant activity was determined using the ferric reducing antioxidant power (FRAP) assay, which was performed as described by Rufino et al. [36], with readings taken at 595 nm using a spectrophotometer (PerkinElmer Lambda 35, Waltham, MA, USA) and a calibration curve of y = 0.0005x − 0.0005 (R^2^ = 0.9995). Ferrous sulfate was used as the standard, and the results were expressed as µM ferrous sulfate/g.

### 2.7. Mineral Composition

Minerals were quantified according to AOAC [37], in which 2 g of dried samples was added into porcelain crucibles. Elemental determinations were performed in triplicate by flame atomic absorption spectrometry using a Varian AA240 spectrometer, (Mulgrave, Victoria, Australia). The concentrations of iron (Fe), zinc (Zn), copper (Cu), manganese (Mn), calcium (Ca), magnesium (Mg), potassium (K), and sodium (Na) were determined. Measurements were initially conducted in the absorbance mode to confirm the linearity of the calibration curves and subsequently in the concentration mode to directly obtain values in mg L^−1^. The precision of the method was established using certified reference values from the standard sample Apple Leaves (No. 1515) provided by the National Institute of Standards and Technology [38].

### 2.8. Diet Formulation

Six diet formulations were prepared for testing, using coproducts from the oil palm production chain, with wheat bran used as the conventional reference diet (Table 1). Considering that the exclusive use of a single coproduct (100%) may result in nutritional imbalances, compromising productivity and larval growth, a combination of wheat bran with different coproducts was adopted [24,39].

For diet preparation, the POME and MF were dried in a forced-air circulation oven (DeLeo TLK48, Porto Alegre-RS, Brazil) at 60 °C for 24 h. Next, the mesocarp fiber was ground using a grain mill (Cadence, MDR302—150 W, São Paulo-SP, Brazil) and sieved (25-mesh). The palm kernel cake was standardized using a 25-mesh sieve.

### 2.9. Larval Rearing

Approximately 115 *Z. atratus* larvae, up to 15 days post hatching, with an average weight of 0.29 ± 0.012 g and an average length of 0.75 ± 0.2 cm, were reared in plastic containers measuring 30 cm × 17 cm × 19 cm (length x height × width), with an opening at the top (19.5 cm × 30 cm) covered with a 25-mesh screen. For each container, diets were added at a ratio of 1:2 (g larvae/g diet), and gauze pads soaked in water were provided and replaced every 2 days as a source of moisture. The containers were placed on shelves and maintained at an average temperature of 25 °C (±1.0 °C) and an average relative humidity of 50% (±5.0%) [16].

On the 13th day of rearing, it was essential to interrupt the experiment due to the progressive increase in mortality in one of the treatments containing only the coproducts from the oil palm industry. Hence, the larvae were separated from the diets and maintained under the same environmental conditions for 24 h to allow gut clearance [16]. The larvae were then washed under running water, dried at room temperature, and frozen in an ultrafreezer at −80 °C (ColdLab CL580-86V, Piracicaba-SP, Brazil). Next, the larvae were freeze-dried (L101 freeze dryer, Liotop, São Paulo-SP, Brazil) for 72 h, and the dried material was ground using a grain mill (Cadence, MDR302—150 W, São Paulo-SP, Brazil) to obtain smaller particles (25-mesh) and stored in an ultrafreezer (ColdLab CL580-86V, São Carlos-SP, Brazil) for further analysis. In the larvae, the crude protein content was determined using a nitrogen-to-protein conversion factor of 4.76 [40], and the carotenoid content was also determined. The analytical methodologies used in this study were similar to those previously described and referenced for the analyses conducted on coproducts from the oil palm production chain.

### 2.10. Larval Growth Performance

The larval growth performance was calculated as described by Zhang et al. [41]. Mass gain was determined as the difference between the initial and final body mass over the rearing period. The efficiency of conversion of ingested food (ECI), feed conversion ratio (FCR), and larval mortality rate (MR) were calculated according to Equations (1)–(3). The average individual feed intake was calculated using the total diet consumption divided by the average number of larvae during the rearing period.

(1) $$ECI\;\left(\%\right)=\left(average\;mass\;gain)÷(average\;individual\;consumption\right)\times 100$$

(2) FCR (g/g)=(average individual consumption)÷(average mass gain)

(3) MR(%)=(number of dead larvae)÷(number of initial larvae)×100

This study was conducted as an exploratory proof-of-concept investigation to assess the effects of using palm oil coproducts as substrates for rearing *Zophobas atratus*, with a focus on observing potential impacts on zootechnical parameters. Therefore, the objective was to generate preliminary evidence rather than to perform formal comparisons among treatments. For this reason, biological replicates for larval performance were not included, and no inferential statistical analyses were performed.

### 2.11. Protein Efficiency Ratio

The protein efficiency ratio (PER) was calculated as described by Sing et al. [42] as the ratio between larval biomass gain and the amount of protein consumed during the experimental period (Equation (4)).

(4) $$PER=\left(Final\;body\;weight-Initial\;body\;weight)÷(Total\;protein\;intake\right)$$

### 2.12. Statistical Analysis

The experimental design was completely randomized. Analyses were conducted in triplicate (*n* = 3), and results were expressed as mean ± standard deviation. Data from diets and larvae were subjected to analysis of variance (ANOVA), and differences were compared using Tukey’s test at a 5% significance level (*p* ≤ 0.05) using the Statistica 8.0 software (Tulsa, Oklahoma, USA).

## 3. Results and Discussion

### 3.1. Physicochemical Characterization of Coproducts

The composition of the coproducts from the oil palm production chain is presented in Table 2, with significant differences (*p* < 0.05) observed among the examined parameters. The chemical composition of oil palm coproducts may vary depending on the species, seasonality, and, moreover, the oil extraction method, which can be performed using either artisanal or industrial techniques. The extraction method directly affects the quality and yield of oil and similarly affects the chemical and nutritional characteristics of the resulting coproducts [1,43,44,45].

The moisture content was highest in the POME (77.03%), followed by mesocarp fiber (42.31%) and palm kernel cake (4.12%). The high moisture level in the effluent is associated with the oil extraction process commonly used in most industries, wherein hot water is added to facilitate phase separation, resulting in a high proportion of residual water that requires appropriate treatment [10]. However, the lower moisture content in palm kernel cake may be advantageous, as increased moisture levels promote microbial growth and product deterioration, consequently reducing its nutritional quality [46].

The crude protein content was higher in palm kernel cake (15.27%) than in the other coproducts. The protein content in palm kernel cake may range from 14% to 21%, depending on the raw material and processing conditions [11,47]. Such levels may be more suitable for animals at less-demanding developmental stages (e.g., adult animals) than for young animals undergoing rapid growth [11]. It has also been reported that palm kernel cake without the fruit shell directly affects the nutritional quality of the coproduct. For instance, Zubaidah et al. [46] reported a higher protein content in the palm kernel cake fraction without the shell (22.3%) than in the fraction with the shell (16.0%), a result attributed to the reduction of indigestible structural components. The presence of fiber, particularly lignocellulose, is associated with lower efficiency in nutrient utilization, including proteins and amino acids [11]. These results emphasize the importance of considering both the coproduct type and processing when investigating its dietary use.

The total lipid content was highest in the POME (17.69%), followed by palm kernel cake (11.57%) and mesocarp fiber (11.04%). The high lipid content of the effluent is associated with the presence of residual oil, estimated at ~4% [48]. This fraction remains after incomplete extraction and separation steps, as free oil and oil adsorbed onto the solid phase [10,49]. The composition of the effluent is strongly affected by operational conditions during the extraction process, such as the pressing efficiency and the use and volume of water, thus exerting a greater effect than plant genetic factors or seasonal variations [44]. This operational variability causes compositional heterogeneity, which may compromise nutritional standardization when the effluent is intended for animal feeding.

In palm kernel cake, lipid contents were similar (11.64–13.19%) to those reported by Amaral-Júnior et al. [50]. Although increased ether extract improves dietary energy density, it may reduce fiber digestibility and forage intake but may not impair productive performance, as demonstrated in buffalo supplemented with this coproduct [50]. These findings indicate that, despite its energetic potential, moderate to high lipid levels may alter nutrient utilization, requiring appropriate dietary balancing.

The carbohydrate content varied significantly among the coproducts, ranging from 3.56% in the POME to 65.68% in the palm kernel cake. The lower value observed in the effluent is related to its high aqueous and lipid fractions. Previous research has indicated that the carbohydrate fraction of palm kernel cake predominantly consists of nonstarch polysaccharides, accounting for ~81% of the total, which is associated with lower digestibility in species with limited capacity to degrade structural carbohydrates [51]. This limitation arises from the low activity of enzymes that can hydrolyze fibrous components, thereby reducing energy utilization. Although palm kernel cake contains monosaccharides, such as glucose, fructose, and mannose, a substantial portion of its carbohydrates is present as structural polysaccharides of the cell wall, which are less accessible to digestion in organisms with low enzymatic activity [11]. In animals such as poultry, this characteristic may limit digestibility and feed efficiency. Nevertheless, dietary supplementation with exogenous enzymes improves the degradation of the fibrous fraction and the nutritional utilization of this coproduct [11,52]. These results demonstrate that the high carbohydrate content in palm kernel cake does not necessarily indicate high energy availability; hence, nutritional evaluation should consider not only total carbohydrate content but also its composition and digestibility.

Palm kernel cake exhibited the highest ash content (3.36%) among the analyzed coproducts. Ash levels in palm kernel cake may range from 3.01% to 7.82% [50]. This variability is also reported by Mollel, Mwangengwa, and Lyimo [53], who described that ash content is essential for the mineral composition of feeds, contributing to bone development, enzymatic function, and overall metabolism.

Palm kernel cake also showed the highest fiber contents, with 68.35% neutral detergent fiber (NDF) and 37.70% acid detergent fiber (ADF). These levels are consistent with the literature and indicate the high lignocellulosic fraction of the coproduct, primarily composed of cellulose, hemicellulose, and lignin [52]. Bamikole and Ikhatua [45] recommended NDF levels of 55–60% and ADF levels of less than 30–32% for ruminants to ensure adequate digestibility and voluntary intake. Therefore, the values observed in the present study indicate that palm kernel cake has a high structural fiber content, which may restrict its direct use in diets, making prior processing or its combination with ingredients of higher energy and protein density necessary.

High fiber levels are associated with reduced carbohydrate digestibility and lower feed efficiency due to the slower degradation rate of the structural fraction and the limited availability of metabolizable energy [54]. Although the controlled inclusion of fibrous ingredients may contribute to fermentative stability in ruminants, the high ADF content, particularly lignin, represents a limiting factor, because this fraction is resistant to enzymatic degradation and reduces overall nutrient utilization [55].

The fatty acid composition (Table 3) differed among the coproducts, indicating their origin within the fruit and the processing stages. Mesocarp fiber displayed a predominance of palmitic acid (C16:0), followed by oleic acid (C18:1n9 cis), a profile characteristic of mesocarp oil. Similarly, the POME exhibited oleic acid as the major component, followed by palmitic acid. This pattern is consistent with previous descriptions of pressed mesocarp oil and effluent, which retain a lipid profile similar to that of crude oil due to incomplete recovery during industrial extraction [56,57]. The high proportions of saturated fatty acids, particularly C16:0, are associated with greater oxidative and thermal stability of palm oil, a vital characteristic for industrial applications. Nevertheless, the predominance of saturated fatty acids may affect lipid digestibility and fat deposition in the consuming organism, depending on the biological system investigated [58].

In contrast, palm kernel cake showed a distinct profile, with a predominance of lauric acid (C12:0), followed by myristic acid (C14:0) and oleic acid (C18:1n9 cis). This profile confers specific physicochemical properties and a differentiated metabolic behavior, which justifies its distinct applications compared with mesocarp coproducts. The literature indicates that variations in extraction method, processing stage, and geographical origin significantly affect the lipid composition of these derivatives [58].

Studies have demonstrated that the inclusion of palm kernel cake in animal diets can modify the fatty acid profile of muscle tissue. For instance, in rabbits, the partial replacement of wheat bran with palm kernel cake resulted in increased fat deposition and higher levels of polyunsaturated fatty acids, without compromising productive performance, in addition to improving the sensory attributes of the meat [59]. Similarly, the inclusion of 25–50% palm fiber (comprising branches, stems, and leaves) in goat diets was associated with a reduction in the levels of saturated fatty acids and an increase in the levels of omega-3 polyunsaturated fatty acids in the meat, without adverse effects on growth [60].

Table 4 shows the mineral contents of oil palm coproducts, with a predominance of potassium, magnesium, sodium, and calcium, among which only sodium did not exhibit significant differences among the coproducts.

A previous study indicated that residues and byproducts from oil palm fruit processing exhibit high concentrations of calcium, magnesium, potassium, copper, and iron, with variations primarily attributed to the plant variety and cultivation conditions [45]. According to that study, sodium content tends to be affected by the plant variety, but not necessarily by the type of residue generated during processing.

From a nutritional viewpoint, minerals play a vital role in metabolic processes, osmotic balance, and enzymatic activity in production animals. Recent research indicates that mineral supplementation in organic form, complexed with amino acids or peptides, can improve productive parameters and the quality of animal-derived products, in addition to contributing to the maintenance of antioxidant capacity. Nevertheless, mineral bioavailability depends on the chemical form and the food matrix [61].

The contents of carotenoids and phenolic compounds and the antioxidant activity of the coproducts are illustrated in Table 5. During palm oil refining, carotenoids are removed to obtain a lighter color; however, their presence in crude oil is associated with greater oxidative stability and antioxidant activity [1]. In the present study, mesocarp fiber showed the highest carotenoid content (138.27 mg/100 g), whereas palm kernel cake had the lowest content (19.94 mg/100 g). The high concentration in the fiber may be attributed to the presence of residual oil, estimated at ~5%, making it 20 times richer in carotenoids than pressed oil [62].

Carotenoid supplementation in poultry diets can improve zootechnical and immunological parameters and affect the visual characteristics of the final product, such as skin and yolk coloration [63]. The inclusion of crude and refined palm oil in the diets of laying hens has been associated with increased levels of β-carotene in the feed, liver, and yolk, promoting improved coloration without affecting productive performance [64]. These data indicate that although carotenoids contribute to functional and antioxidant properties, their effects on performance depend on the species and the level of inclusion.

In the present study, palm kernel cake showed the highest content of phenolic compounds (7.27 mg/g), a value greater than that previously reported for this coproduct (5.19 mg/g) and for pressed fiber (3.59 mg/g) [65]. The negative correlation observed between total phenolics and the DPPH assay, together with the positive correlation with FRAP, suggests a greater reducing capacity of palm kernel cake, a pattern also described in recent research [66]. Thus, the high FRAP value for palm kernel cake can be attributed to the presence of phenolic compounds such as pyrogallol, 4-hydroxybenzoic acid, gallic acid, and ferulic acid, which exhibit protective bioactivity against cardiovascular diseases, without toxicity or teratogenic effects [67].

Dietary polyphenols have been associated with improved productive performance and feed efficiency in poultry and swine, possibly through the modulation of the intestinal microbiota, increased digestibility, and stimulation of endogenous antioxidant activity [68,69]. Nevertheless, these effects are dose-dependent, as high levels may reduce palatability and voluntary intake. Therefore, although palm kernel cake demonstrates potential as a natural source of antioxidants, it is necessary to consider the balance between bioactivity and acceptability for its dietary application.

### 3.2. Larval Growth Performance

During the evaluation of larval growth performance, the experiment was interrupted on day 13 of rearing due to the progressive increase in mortality in the treatment containing diet D5 (33.33% mesocarp fiber +33.33% palm oil mill effluent + 33.33% palm kernel cake). The decision to terminate the experiment prematurely was necessary due to the risk of insufficient larval biomass for the planned analyses and for maintaining comparability with the other diets, as well as to enable the refinement of formulation and processing strategies in subsequent studies.

Certain organic residues may exert adverse effects on the larvae of *Tenebrio molitor*, a species from the same family as *Z. atratus*. Low growth and high mortality in *T. molitor* fed on tomato residues combined with wheat bran have been detected, although without clarifying the underlying mechanisms [70]. Moreover, plant residues have been associated with reduced feed conversion efficiency, alterations in body composition, and increased mortality in *T. molitor* and *Z. atratus* [71,72].

**Figure 1:**
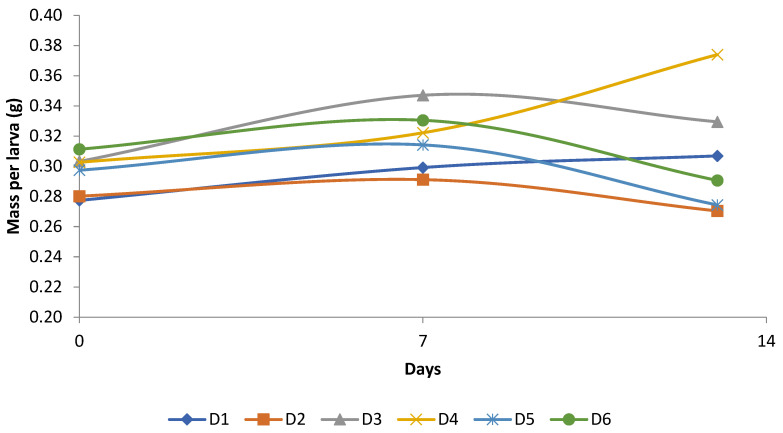
Average mass (grams) of *Zophobras atratus* larvae fed a conventional diet with or without coproducts from the oil palm production chain. WB: Wheat bran; MF: Mesocarp fiber; POME: Palm oil mill effluent; PKC: Palm kernel cake. Diets: D1: 100% WB; D2: 50% WB and 50% MF; D3: 50% WB and 50% POME; D4: 50% WB and 50% PKC; D5: 33.33% MF, 33.33% POME and 33.33% PKC; D6: 33.33% WB, 33.33% MF and 33.33% POME.

Considering the previously presented composition, oil palm coproducts are characterized by high lipid content, a fibrous fraction, and the presence of phenolic compounds. Larvae with low growth performance were fed on D2, D3, D5, and D6, which may be associated with the presence of nutritional factors exerting negative effects (Figure 1). In insects of the family Tenebrionidae, larval growth is highly dependent on the protein/energy balance, and diets with excess lipids or protein deficiency tend to impair feed conversion efficiency and larval growth, possibly due to metabolic limitations [73,74,75]. Furthermore, plant secondary metabolites, including polyphenols, may interact with proteins and digestive enzymes, thus reducing digestibility and affecting growth performance and mortality [76,77].

Despite the recorded mortality, all diets promoted mass gain during the first week (D1: 7.83%; D2: 3.92%; D3: 14.44%; D4: 6.45%; D5: 5.62%; D6: 6.18%), indicating that the substrates provided initial nutritional availability (Figure 1). However, in the second week, mass loss was observed in most treatments, except for D1 (2.59%) and D4 (16.08%), suggesting that the maintenance of growth depends not only on the initial composition but also on the stability and preservation of the substrate over time. The lack of diet renewal may have favored the oxidative deterioration of lipids and the accumulation of toxic metabolites, thereby reducing larval performance, an effect reported previously in rearing systems using agro-industrial residues [71,72].

Palm kernel cake has been investigated as an alternative ingredient for poultry, swine, and ruminants, with results depending on the level of inclusion and enzymatic supplementation [11,78,79]. In insect production, oil palm coproducts also demonstrate potential, although with certain limitations. In the larvae of *Hermetia illucens*, the inclusion of palm decanter cake resulted in a growth rate of 0.23 g/day, a significant weight gain, up to day 11, and the onset of the pupal stage from day 12 onward [80]. The authors of that study observed that the development time varied according to the substrate quality, being shorter in nutritionally more adequate diets, reinforcing the direct impact of residue composition on the biological cycle.

Lim et al. [81] also used palm decanter cake to feed *H. illucens* larvae; however, the residue was treated with the enzyme cellulase to facilitate the breakdown of cellulose into glucose, thereby increasing its assimilation by the larvae. They reported that after 16 days of rearing and with increased glucose concentration in the diet, there was improved larval growth performance, reaching 6.56 mg/larva. This result suggests that improved carbohydrate availability increases assimilation and growth, indicating that the limitation does not lie in the raw material itself but in its metabolic availability.

The daily mass gain of the larvae (Table 6) confirmed that initial performance was more prominent during the first week, with D4 resulting in growth similar to that with control (D1), whereas D2, D3, and D6 resulted in initial growth, but this pattern was not sustained. Nascimento et al. [19] investigated growth performance in *Z. atratus* larvae fed on winery residue and reported that the highest daily mass gain occurred during the initial period and may be related to a greater nutrient demand. Conversely, in the later stages of rearing, the accumulation of dietary residues in the substrate and the deterioration of the diet itself may impair larval performance and increase adverse effects, possibly associated with the formation of toxic compounds. This interpretation is consistent with the findings of the present study, because no diet renewal was performed throughout the experiment.

Larvae fed on D5 exhibited unsatisfactory performance from the first days of rearing, which may be related to the fact that this diet consisted exclusively of coproducts, without the addition of wheat bran. According to Montalbán et al. [39], diets formulated solely using residues or coproducts tend to negatively affect larval development due to possible nutritional imbalances and limitations in substrate digestibility. In *T. molitor* larvae, the ingestion of diets with high NDF content exerts a negative impact on nutrient assimilation, feed conversion efficiency, and weight gain [82]. Moreover, nutritionally unbalanced diets may adversely affect the microbiota associated with the digestive tract, with direct consequences for productive performance, as demonstrated in *H. illucens* larvae [83].

Table 7 shows the efficiency of conversion (ECI), feed conversion ratio (FCR), and mortality rate (MR), wherein ECI indicates how much of the ingested diet was converted into animal biomass, and FCR represents the amount of feed the larvae must consume to obtain 1 kg of biomass, which is more efficient at lower FCR values [19].

The highest ECI and the lowest FCR values were observed in larvae fed on wheat bran supplemented with palm kernel cake (D4), indicating greater feed utilization efficiency in this treatment. This was followed by larvae fed exclusively on the control diet (D1) and those fed on diet containing wheat bran and POME (D3). Nevertheless, among these three treatments, the inclusion of POME resulted in the highest larval mortality rate (29.57%), possibly associated with the high lipid content of this coproduct, which may compromise the survival and performance of the larvae. The reduction in survival rate may also be related to decreased enzymatic activity, hormonal performance, and nutrient absorption in the larvae [84].

The higher efficiency observed with D4 may be attributed to the higher protein content of palm kernel cake (22.25%, dry matter basis), which favors larval protein synthesis. In *T. molitor*, diets with higher protein content (~20.4%) resulted in improved feed conversion (50.1%), reinforcing the importance of adequate protein supply to maximize ECI [85]. The efficiency of amino acid incorporation into body biomass is a key determinant of growth, weight gain, and reduction of catabolism [16].

In the remaining treatments, negative ECI and FCR values indicate nutritional constraints of the substrate and loss of body mass. This result suggests energy limitation or excess lipid content, factors that reduce digestibility and compromise nutrient utilization efficiency. An imbalance in the protein/carbohydrate ratio may result in inefficient amino acid catabolism when energy availability is insufficient, leading to reduced protein deposition [86,87]. These mechanisms may explain the reduced growth and lower feed efficiency observed with these diets, as well as the presence of residual tannins from processing, which can interact with proteins and digestive enzymes, reducing digestibility [88].

### 3.3. Nutritional Composition of Larvae

As shown in Table 8, larvae fed on D4 (50% wheat bran + 50% palm kernel cake) demonstrated the highest body protein content (39.14%). This treatment also resulted in the best growth performance, which may be attributed to the inclusion of palm kernel cake, a coproduct that exhibited a balanced nutrient profile and the highest crude protein content (15.27%) among the investigated residues. Experimental data demonstrate that substrates with higher protein content promote an increase in protein levels in larval biomass, as they facilitate the ingestion, metabolism, and conversion of amino acids into body protein [16,39]. The larvae of *T. molitor* fed on substrates with higher protein content (broccoli byproduct) demonstrated greater final weight and improved productive performance, thus reinforcing the importance of adequate protein supply [39]. Although palm kernel cake exhibited the highest content of phenolic compounds among the investigated residues, there was no impairment in protein deposition or larval growth. Phenolic compounds may form complexes with proteins, thus reducing digestibility [23]. Nevertheless, the results suggest that, under the tested conditions, the levels of these compounds were insufficient to limit protein utilization. This indicates that dietary balance, rather than the isolated presence of specific compounds, determines performance.

Regarding carotenoids, diets D2 and D6 promoted the highest larval contents, with values exceeding those of the control by >193%. This increase is consistent with the high concentration of carotenoids in the mesocarp fiber, demonstrating their transfer to larval tissues. In *H. illucens* larvae, increases of >230% in carotenoid content were observed when larvae were fed on byproducts rich in these compounds, such as tomatoes and carrots [89], thus corroborating the bioaccumulation capacity in insects.

The protein and carotenoid contents observed in *Z. atratus* larvae indicate that this species has potential as a nutritional and functional ingredient in animal feed, particularly for monogastric species. The presence of carotenoids in the larvae improves their functional value, because these compounds are associated with antioxidant activity, immune support, and, especially, the pigmentation of animal-derived products, a relevant characteristic for laying hens and broilers, in which yolk, skin, or meat coloration represents an important commercial quality attribute [63].

In this context, it is essential to evaluate protein quality to determine the viability of using biomass in dietary formulations, such as the protein efficiency ratio (PER), which expresses the relationship between body mass gain and the amount of protein consumed, reflecting in an integrated way the digestibility and the capacity for protein deposition in the organism, using casein as a reference protein [90]. In this study, only the larvae fed the control diet (D1) and the diet supplemented with palm kernel cake (D4) showed positive PER values, 2.82 and 1.34, respectively. Values higher than those of casein (1.22) reinforced the applicability of the protein efficiency ratio as a tool to evaluate the nutritional potential of these biomasses in animal production systems, indicating that *Z. atratus* has the potential to be a sustainable protein source of the future.

As the nutritional composition of larvae is strongly modulated by diet and determines their suitability as an ingredient for different production chains, it is necessary to define the final use of the larval biomass in order to direct its nutritional quality and zootechnical application. In this context, *Zophobas atratus* larval biomass can be proposed as a protein ingredient for fish and poultry feeds, as well as for applications in industrial pet food and as a source of lipids and bioactive compounds.

Overall, this study demonstrates that the incorporation of oil palm coproducts into the diet of *Z. atratus* is directly conditioned by the digestibility of the fibrous fraction and an adequate protein/energy balance. The variable nutritional composition of these coproducts, particularly palm kernel cake, indicates that the inclusion of residues alone does not guarantee satisfactory performance. The combination of wheat bran with palm kernel cake promoted larval growth and maintained adequate conversion efficiency, supporting that dietary protein content directly affects body protein deposition. Therefore, protein adjustment and, when required, pretreatment of the fibrous fraction may be vital strategies to maximize performance and enable the use of these residues in bioconversion systems.

Protein availability influences larval growth and composition, with an optimal protein threshold for maximum conversion efficiency. In addition, the ability of the gut microbiota to degrade fiber promotes growth and protein deposition, reinforcing that the effective utilization of these coproducts depends on proper nutritional balancing and efficient use of the fibrous fraction [91].

The variable nutritional composition of these coproducts, particularly palm kernel cake, indicates that the inclusion of residues alone does not guarantee satisfactory performance. The combination of wheat bran with palm kernel cake promoted larval growth and maintained adequate conversion efficiency, corroborating the notion that the dietary protein level directly affects body protein deposition.

Coproducts such as POME may contain antinutritional compounds that contribute to the mortality observed in the tested formulations, reinforcing the need for appropriate technological treatment, such as enzyme application, before inclusion in *Z. atratus* diets. Therefore, protein adjustment and, when necessary, physical, chemical, or enzymatic pretreatment of the fibrous fraction may be vital strategies to maximize performance and enable the use of these residues in bioconversion systems.

The findings of this study provide relevant preliminary evidence for the design of future studies with optimized formulations, longer experimental duration, and a robust statistical design.

## 4. Conclusions

The nutritional composition of coproducts from the oil palm production chain investigated in this study varies according to different factors related to extraction and processing methods. The highest contents of protein, carbohydrates, fiber, and saturated fatty acids were found in palm kernel cake, which also demonstrated greater antioxidant capacity. The mesocarp fiber exhibited the highest carotenoid content, whereas the POME showed the highest lipid content. This composition confirms that the nutritional and functional value of these coproducts depends directly on their chemical characteristics, with implications for animal feeding, including edible insects.

Nevertheless, the hypothesis that partial replacement of the conventional substrate with oil palm coproducts would maintain the productive performance of *Z. atratus* was only partially confirmed. Although the inclusion of palm kernel cake improved conversion parameters and body composition, all formulations containing coproducts resulted in mortality, indicating that, under the investigated conditions, these coproducts did not completely meet the nutritional requirements of the species.

However, the mortality observed in treatments with higher proportions of byproducts demonstrates that their direct inclusion still presents important limitations, probably associated with the high fibrous fraction, lipid content, antinutritional compounds, and nutritional imbalance of the diets. Therefore, further studies are needed to optimize formulation strategies, improve the protein/energy balance, and evaluate physical, chemical, or enzymatic pretreatments capable of increasing digestibility and nutrient availability. Even with these limitations, the results presented provide an important direction for future studies and corroborate the potential use of palm oil by-products as promising resources for insect bioconversion systems, aligned with the circular economy and sustainable waste valorization approaches.

## Figures and Tables

**Table 1:** Diet formulations for feeding *Zophobas atratus*.

Raw Material	Diets ^1^					
	D1	D2	D3	D4	D5	D6
Wheat bran (WB)	100	50	50	50	0	33.33
Mesocarp fiber (MF)	0	50	0	0	33.33	33.33
Palm oil mill effluent (POME)	0	0	50	0	33.33	33.33
Palm kernel pie (PKC)	0	0	0	50	33.33	0

^1^ Values in mass/mass percentage.

**Table 2:** Proximate composition, NDF, ADF, and energy value of coproducts from the oil palm production chain.

Parameters	Coproducts ^1^		
	MF	POME	PKC
Moisture (%)	42.31 ± 0.01 ^b^	77.03 ± 0.07 ^a^	4.12 ± 0.01 ^c^
Protein (%)	5.77 ± 0.05 ^b^	1.44 ± 0.03 ^c^	15.27 ± 0.01 ^a^
Lipids (%)	11.04 ± 0.06 ^b^	17.69 ± 0.04 ^a^	11.57 ± 0.03 ^b^
Carbohydrates (%)	38.44 ± 0.01 ^b^	3.56 ± 0.05 ^c^	65.68 ± 0.05 ^a^
Ash (%)	2.44 ± 0.02 ^b^	0.28 ± 0.04 ^c^	3.36 ± 0.05 ^a^
NDF (%)	34.08 ± 0.01 ^b^	2.05 ± 0.01 ^c^	68.35 ± 0.06 ^a^
ADF (%)	22.22 ± 0.05 ^b^	1.74 ± 0.06 ^c^	37.70 ± 0.03 ^a^
Energy (Kcal/100 g)	276.20 ± 0.40 ^b^	179.21 ± 0.10 ^c^	427.93 ± 0.60 ^a^

^1^ Values expressed as arithmetic mean and standard deviation of samples on a wet basis. MF: Mesocarp fiber; POME: Palm oil mill effluent; PKC: Palm kernel cake; NDF: Neutral detergent fiber; ADF: Acid detergent fiber. Different letters in the same row indicate significant differences by Tukey’s test (*p* ≤ 0.05; *n* = 3).

**Table 3:** Fatty acid profile of coproducts from the oil palm production chain.

Fatty Acids (%)	Coproducts		
	MF	POME	PKC
Saturated			
C8:0	ND	ND	3.81
C10:0	ND	ND	3.32
C12:0	0.17	0.35	48.82
C14:0	0.47	0.68	16.32
C16:0	46.74	37.99	8.28
C18:0	7.50	5.77	2.51
Monounsaturated			
C18:1n9 cis	38.73	45.68	14.43
Polyunsaturated			
C18:2n6 cis	6.40	9.52	2.49
ΣSFA	54.88	44.79	83.06
ΣMUFA	38.73	45.68	14.43
ΣPUFA	6.40	9.52	2.49

MF: Mesocarp fiber; POME: Palm oil mill effluent; PKC: Palm kernel cake; ND: Not detected; ΣSFA: Sum of saturated fatty acids; ΣMUFA: Sum of monounsaturated fatty acids; ΣPUFA: Sum of polyunsaturated fatty acids.

**Table 4:** Mineral content of coproducts from the oil palm production chain.

Minerals (mg/100 g)	MF	POME	PKC
Iron	18.80 ± 0.23 ^b^	18.87 ± 0.05 ^b^	26.09 ± 0.02 ^a^
Zinc	0.93 ± 0.11 ^b^	0.30 ± 0.05 ^b^	2.69 ± 0.02 ^a^
Copper	0.29 ± 0.02 ^b^	0.10 ± 0.02 ^c^	0.85 ± 0.04 ^a^
Manganese	1.31 ± 0.01 ^b^	1.11 ± 0.02 ^c^	5.97 ± 0.01 ^a^
Calcium	65.45 ± 0.40 ^a^	43.28 ± 0.06 ^b^	23.51 ± 0.94 ^c^
Magnesium	100.33 ± 3.27 ^a^	93.53 ± 0.57 ^c^	96.46 ± 0.85 ^b^
Potassium	345.79 ± 8.77 ^a^	321.03 ± 6.94 ^b^	251.95 ± 7.62 ^c^
Sodium	96.17 ± 4.65 ^a^	96.26 ± 14.13 ^a^	96.12 ± 15.81 ^a^

Values expressed as arithmetic mean and standard deviation. MF: Mesocarp fiber; POME: Palm oil mill effluent; PKC: Palm kernel cake. Different letters in the row indicate significant differences by Tukey’s test (*p* ≤ 0.05; *n* = 3).

**Table 5:** Total carotenoids and antioxidant activity of coproducts from the oil palm production chain.

Parameters	Coproducts ^1^		
	MF	POME	PKC
Carotenoids (mg/100 g)	138.27 ± 0.21 ^a^	32.10 ± 0.10 ^b^	19.94 ± 0.08 ^c^
Total phenolics (mg GAE/g)	6.01 ± 0.05 ^b^	3.74 ± 0.04 ^c^	7.27 ± 0.03 ^a^
DPPH (g/g DPPH)	3.35 ± 0.06 ^a^	3.53 ± 0.05 ^a^	1.51 ± 0.04 ^b^
FRAP (µM ferrous sulfate/g)	8.01 ± 0.07 ^b^	7.12 ± 0.03 ^b^	25.08 ± 0.02 ^a^

^1^ Values expressed as arithmetic mean and standard deviation. MF: Mesocarp fiber; POME: Palm oil mill effluent; PKC: Palm kernel cake. Different letters in the row indicate significant differences by Tukey’s test (*p* ≤ 0.05; *n* = 3).

**Table 6:** Daily weight gain of *Zophobas atratus* larvae fed on a conventional diet compared with diets containing byproducts from the oil palm production chain.

Mass Gain (g/Day)	Period (Days)	Diets					
		D1	D2	D3	D4	D5	D6
DMG 1	0 to 7	0.3571	0.1805	0.0751	0.3205	−0.5330	0.0325
DMG 2	7 to 13	0.1483	−0.6679	−1.4517	−0.1916	−2.7470	−2.2743

DMG 1: average daily weight gain of larvae divided by the first week of rearing; DMG 2: average daily weight gain of larvae divided by the second week of rearing. WB: Wheat bran; MF: Mesocarp fiber; POME: Palm oil mill effluent; PKC: Palm kernel cake. Diets: D1: 100% WB; D2: 50% WB and 50% MF; D3: 50% WB and 50% POME; D4: 50% WB and 50% PKC; D5: 33.33% MF, 33.33% POME and 33.33% PKC; D6: 33.33% WB, 33.33% MF and 33.33% POME.

**Table 7:** Conversion efficiency parameters in larval biomass and mortality rate of *Zophobas atratus* larvae fed on coproducts from the oil palm production chain.

Diets	D1	D2	D3	D4	D5	D6
ECI (%)	17.55	−17.97	13.23	78.99	−22.37	−24.99
FCR (%)	4.40	−5.46	8.75	0.90	−5.19	−4.49
MR (%)	0.00	5.22	29.57	16.52	55.65	33.04

ECI: Feed conversion efficiency (dry basis); FCR: Feed conversion ratio (wet basis); MR: Mortality rate. WB: Wheat bran; MF: Mesocarp fiber; POME: Palm oil mill effluent; PKC: Palm kernel cake. Diets: D1: 100% WB; D2: 50% WB and 50% MF; D3: 50% WB and 50% POME; D4: 50% WB and 50% PKC; D5: 33.33% MF, 33.33% POME and 33.33% PKC; D6: 33.33% WB, 33.33% MF and 33.33% POME.

**Table 8:** Protein and carotenoid contents in *Zophobas atratus* larvae fed on coproducts from the oil palm production chain.

Larval Proteins	Proteins (%)	Carotenoids (mg/100 g)
D1	35.33 ± 0.67 ^c^	18.18 ± 0.74 ^e^
D2	37.98 ± 0.41 ^b^	35.19 ± 3.27 ^a^
D3	35.91 ± 0.23 ^c^	28.49 ± 1.39 ^d^
D4	39.14 ± 0.41 ^a^	30.45 ± 0.37 ^c^
D5	34.61 ± 0.31 ^c^	32.37 ± 3.03 ^b^
D6	32.61 ± 0.34 ^d^	35.20 ± 0.78 ^a^

Values expressed as arithmetic mean and standard deviation. Different letters in the column indicate significant differences by Tukey’s test (*p* ≤ 0.05; *n* = 3). WB: Wheat bran; MF: Mesocarp fiber; POME: Palm oil mill effluent; PKC: Palm kernel cake. Diets: D1: 100% WB; D2: 50% WB and 50% MF; D3: 50% WB and 50% POME; D4: 50% WB and 50% PKC; D5: 33.33% MF, 33.33% POME and 33.33% PKC; D6: 33.33% WB, 33.33% MF and 33.33% POME.

## Data Availability

The data presented in this study are available on request from the corresponding authors. The data are not publicly available because the datasets are stored in institutional archives and are available upon request.

## Abbreviations

The following abbreviations are used in this manuscript:

ADF	acid detergent fiber
ANOVA	analysis of variance
DOI	digital object identifier
ECI	efficiency of conversion of ingested
FAME	fatty acid methyl esters
FCR	feed conversion ratio
FRAP	ferric reducing antioxidant power
MF	mesocarp fiber
MR	mortality rate
NDF	neutral detergent fiber
NIST	National Institute of Standards and Technology
PKC	palm kernel cake
POME	palm oil mill effluent
SFA	saturated fatty acids

## References

[1] Alhaji A.M., Almeida E.S., Carneiro C.R., Silva C.A.S., Monteiro S., Coimbra J.S.R. (2024). Palm oil (*Elaeis guineensis*): A journey through sustainability, processing, and utilization. Foods.

[2] Mordor Intelligence Palm Oil Market—Size, Share and Trends Analysis. https://www.mordorintelligence.com/industry-reports/palm-oil-market.

[3] United States Department of Agriculture (USDA) (2025). Oilseeds: World Markets and Trade.

[4] Kahar P., Rachmadona N., Pangestu R., Palar R., Adi D.T.N., Juanssilfero A.B., Yopi, Manurung I., Hama S., Ogino C. (2022). An integrated biorefinery strategy for the utilization of palm oil wastes. Bioresour. Technol..

[5] Meijaard E., Brooks T.M., Carlson K.M., Slade E.M., Garcia-Ulloa J., Gaveau D.L., Lee J.S.H., Santika T., Juffe-Bignoli D., Struebig M.J. (2020). The environmental impacts of palm oil in context. Nat. Plants.

[6] Hau E.H., Teh S.S., Yeo S.K., Chua B.L., Mah S.H. (2022). Transformation of oil palm biomass into value-added components. Rev. Agric. Sci..

[7] Awere E., Bonoli A., Obeng P.A. (2020). Solids–liquid separation and solar drying of palm oil mill wastewater sludge: Potential for sludge reuse. Case Stud. Chem. Environ. Eng..

[8] Hora J.A.S.F. (2023). Characterization of Residues from Palm Oil (“Dendê Oil”) Production and Their Final Disposal in Valença, BA. Master’s Thesis.

[9] Gamarra-Romero L.F., Mora H.E.G., Cipra-Rodriguez J.A.C., Cárdenas-Oscanoa A.J.C. (2024). Effect of adding oil palm (*Elaeis guineensis* Jacq.) mesocarp fibers to cement composites. Colomb. For..

[10] Mohammad S., Baidurah S., Kobayashi T., Ismail N., Leh C.P. (2021). Palm oil mill effluent treatment processes—A review. Processes.

[11] Azizi M.N., Loh T.C., Foo H.L., Chung E.L.T. (2021). Is palm kernel cake a suitable alternative feed ingredient for poultry?. Animals.

[12] Rosa M.F., Souza Filho M.S.M., Figueiredo M.C.B., Morais J.P.S., Santaella S.T., Leitão R.C. Valorization of agro-industrial residues. Proceedings of the II International Symposium on Agricultural and Agro-Industrial Waste Management (SIGERA).

[13] Oliveira J., Furlan Júnior J., Teixeira L.B. (2006). Chemical Composition of Boiler Ash from the Oil Palm Agroindustry.

[14] Ferreira W.A., Botelho S.M., Vilar R.R.L. (1998). Chemical Composition of Oil Palm Agro-Industrial by-Products.

[15] Machado S.S.N., da Silva J.B.A., Nascimento R.Q., Lemos P.V.F., de Jesus Assis D., Marcelino H.R., de Souza Ferreira E., Cardoso L.G., Pereira J.D., Santana J.S. (2024). Insect residues as an alternative and promising source for the extraction of chitin and chitosan. Int. J. Biol. Macromol..

[16] Tavares P.P.L.G., Lima M.S., Boa Morte E.S., Bulos R.B.A., Ribeiro C.V.M., Souza C.O. (2025). Replacing conventional substrate with linseed cake improves the omega-3 profile of *Zophobas atratus* Fabricius (Coleoptera: Tenebrionidae) larvae. Future Foods.

[17] Do Prado N.B., Lemos P.V.F., de Andrade A.B., Pereira J.D., Grisi C.V.B., Nascimento M.B., Mesquita P.R.R., de Magalhães Cordeiro A.M.T., de Almeida Lucas A., da Silva J.B.A. (2025). From larvae to oil: Physicochemical characterization and thermo-oxidative stability of lipids extracted from *Zophobas atratus*. Food Chem..

[18] Hancz C., Sultana S., Nagy Z., Biró J. (2024). The role of insects in sustainable animal feed production for environmentally friendly agriculture: A review. Animals.

[19] Nascimento R.Q., Di Mambro Ribeiro C.V., Colauto N.B., da Silva L., Lemos P.V.F., de Souza Ferreira E., Linde G.A., Machado B.A.S., Tavares P.P.L.G., Biasoto A.C.T. (2022). Utilization of agro-industrial residues in the rearing and nutritional enrichment of *Zophobas atratus* larvae: New food raw materials. Molecules.

[20] Siddiqui A.S., Harahap I.A., Osei-Owusu J., Saikia T., Wu Y.S., Fernando I., Perestrelo R., Câmara J.S. (2024). Bioconversion of organic waste by insects—A comprehensive review. Process Saf. Environ. Prot..

[21] Eggink K.M., Lund I., Pedersen P.B., Hansen B.W., Dalsgaard J. (2022). Biowaste and by-products as rearing substrates for black soldier fly (*Hermetia illucens*) larvae: Effects on larval body composition and performance. PLoS ONE.

[22] Bacelar T.V.M., de Souza C.O., Ayena K., Moraes C.P.M., Nascimento R.Q., Biasoto A.C.T., Umsza-Guez M.A. (2026). Effects of a wine lees-based diet on the growth, antioxidant status, nutritional and phenolic composition of *Zophobas atratus* larvae. Eur. Food Res. Technol..

[23] Ruschioni S., Loreto N., Foligni R., Mannozzi C., Raffaelli N., Zamporlini F., Pasquini M., Roncolini A., Cardinali F., Osimani A. (2020). Addition of olive pomace to feeding substrate affects growth performance and nutritional value of mealworm (*Tenebrio molitor* L.) larvae. Foods.

[24] Fasce B., Ródenas L., López M.C., Moya V.J., Pascual J.J., Cambra-López M. (2022). Nutritive value of wheat bran diets supplemented with fresh carrots and wet brewers’ grains in yellow mealworm. J. Insect Sci..

[25] AOAC (2019). Official Methods of Analysis of AOAC International.

[26] Agência Nacional de Vigilância Sanitária (ANVISA) (2003). Resolution RDC No. 360, of 23 December 2003—Technical Regulation on Nutrition Labeling of Packaged Foods.

[27] Atwater W.O., Woods C.D. (1896). The Chemical Composition of American Food Materials.

[28] Van Soest P.J., Robertson J.B., Lewis B.A. (1991). Methods for dietary fiber, neutral detergent fiber, and nonstarch polysaccharides in relation to animal nutrition. J. Dairy Sci..

[29] Bligh E.G., Dyer W.J. (1959). A rapid method of total lipid extraction and purification. Can. J. Biochem. Physiol..

[30] Rodriguez-Amaya D.B. (2001). A Guide to Carotenoid Analysis in Foods.

[31] Wondracek D.C., Vieira R.F., Silva D.B., Agostini-Costa T.S., Sano S.M., Faleiro F.G. (2012). Influence of saponification on the determination of carotenoids in cerrado passion fruits. Quim. Nova.

[32] Oliver J., Palou A. (2000). Chromatographic determination of carotenoids in foods. J. Chromatogr. A.

[33] Castro P.A.C. (2019). Extraction of Phenolic Compounds from Oil Palm Mesocarp Cake [Trabalho de Conclusão de Curso]. Bachelor’s Thesis.

[34] Singleton V.L., Rossi J.A. (1965). Colorimetry of total phenolics with phosphomolybdic–phosphotungstic acid reagents. Am. J. Enol. Vitic..

[35] Rufino M.S.M., Alves R.E., Brito E.S., Morais S.L., Sampaio C.G., Pérez-Jiménez J., Saura-Calixto F.D. (2007). Scientific Methodology: Determination of Total Antioxidant Activity in Fruits Using DPPH Free Radical Scavenging Method.

[36] Rufino M.S.M., Alves R.E., Brito E.S., Morais S.L., Sampaio C.G., Pérez-Jiménez J., Saura-Calixto F.D. (2006). Scientific Methodology: Determination of Total Antioxidant Activity in Fruits by the Ferric Reducing Antioxidant Power (FRAP) Method.

[37] AOAC (2012). Official Methods of Analysis of AOAC International.

[38] National Institute of Standards and Technology (NIST) (2022). Certificate of Analysis: Standard Reference Material 1515—Apple Leaves.

[39] Montalbán A., Martínez-Miró S., Schiavone A., Madrid J., Hernández F. (2023). Growth performance, diet digestibility, and chemical composition of mealworm (*Tenebrio molitor* L.) fed agricultural by-products. Insects.

[40] Janssen R.H., Vincken J.P., Van Den Broek L.A.M., Fogliano V., Lakemond C.M.M. (2017). Nitrogen-to-protein conversion factors for three edible insects: *Tenebrio molitor*, *Alphitobius diaperinus*, and *Hermetia illucens*. J. Agric. Food Chem..

[41] Zhang X., Tang H., Chen G., Qiao L., Li J., Liu B., Liu Z., Li M., Liu X. (2019). Growth performance and nutritional profile of mealworms reared on corn stover, soybean meal, and distillers’ grains. Eur. Food Res. Technol..

[42] Sing K.W., Kamarudin M.S., Wilson J.J., Sofian-Azirun M. (2014). Evaluation of blowfly (*Chrysomya megacephala*) maggot meal as an effective, sustainable replacement for fishmeal in the diet of farmed juvenile red tilapia (*Oreochromis* sp.). Pak. Vet. J..

[43] Mahlia T.M.I., Ismail N., Hossain N., Silitonga A.S., Shamsuddin A.H. (2019). Palm oil and its wastes as bioenergy sources: A comprehensive review. Environ. Sci. Pollut. Res..

[44] Fonseca L.S., Campanha R.B., Oliveira M.E.C., Mendonça S. Chemical and physical characterization of industrial residues from oil palm tenera and hybrid Manicoré. Proceedings of the III Encontro de Pesquisa e Inovação da Embrapa Agroenergia.

[45] Bamikole M.A., Ikhatua U.J. (2009). Variety diversity effect on the chemical composition and dry matter degradation characteristics of residue and by-products of oil palm fruits. Anim. Sci. J..

[46] Zubaidah S., Hanim C., Ariyadi B., Baskara A.J., Zuprizal (2024). Nutrient composition and cell-wall structure of palm kernel cake supplemented with enzymes. Adv. Anim. Vet. Sci..

[47] Putri E.M., Zain M., Warly L., Hermon H. (2019). In vitro evaluation of ruminant feed from West Sumatera based on chemical composition and content of rumen degradable and rumen undegradable proteins. Vet. World.

[48] Ong E.S., Rabbani A.H., Habashy M.M., Abdeldayem O.M., Al-Sakkari E.G., Rene E.R. (2021). Palm oil industrial wastes as a promising feedstock for biohydrogen production: A comprehensive review. Environ. Pollut..

[49] Wan Sharifudin W.S.S.A., Sulaiman A., Mokhtar N., Baharuddin A.S., Tabatabaei M., Busu Z., Subbian K. (2015). Presence of residual oil in relation to solid particle distribution in palm oil mill effluent. BioResources.

[50] do Amaral-Júnior J.M., de Morais E., Lima A.C.S., Martorano L.G., de Souza Nahúm B., Sousa L.F., de Brito Lourenço-Júnior J., de Carvalho Rodrigues T.C.G., da Silva J.A.R., da Costa Silva A.L. (2023). Effect of palm kernel cake supplementation on voluntary feed intake, in situ rumen degradability and performance in buffaloes in the Eastern Amazon. Animals.

[51] Silva R.S., Santo R.V.E., Barbosa A.V.C., Santos M.A.S., Corrêa R.O., Martins Júnior H., Lpurenço Júnior J.B. (2019). Apparent digestibility of palm kernel meal to tambaqui (*Colossoma macropomum*). Arq. Bras. Med. Vet. Zootec..

[52] Azizi M.N., Loh T.C., Chung E.L.T., Aziz M.F.A., Foo H.L., Liu J., Farzana Z.A., Raj L.S. (2025). From nutritional profiles to digestibility insights: Exploring palm kernel cake and decanter cake in broiler diets. Animals.

[53] Mollel E.D., Mwangengwa L., Lyimo C.M. (2025). Nutritional and digestibility profile of palm kernel cake (*Elaeis guineensis*) produced in different regions of Tanzania. Int. J. Anim. Sci. Technol..

[54] García H.G., Rodriguez A.A., Bejarano J.C.E., SanMiguel E.G., Licón C.H., Sánchez-Verín C.V. (2007). Effect of alfalfa supplementation on volatile fatty acid concentration and pH in the rumen of steers fed a basal diet of wheat straw. Cienc. En Front. Rev. Cienc. Y Tecnol. UACJ.

[55] Fhonna F.A., Jayanegara A., Wajizah S., Samsudin A.A., Samadi S. (2025). A meta-analysis on the digestibility of fiber and non-fiber carbohydrates in small ruminants consuming palm kernel cake. IOP Conf. Ser. Earth Environ. Sci..

[56] Teh S.S., Lau H.L.N. (2021). Quality assessment of refined red palm-pressed mesocarp olein. Food Chem..

[57] Muanruksa P., Winterburn J., Kaewkannetra P. (2021). Biojet fuel production from waste of palm oil mill effluent through enzymatic hydrolysis and decarboxylation. Catalysts.

[58] Afifi E.H., John Martin J.J., Wang Q., Li X., Liu X., Zhou L., Li R., Fu D., Li Q., Ye J. (2025). Fatty acid and lipid metabolism in oil palm: From biochemistry to molecular mechanisms. Int. J. Mol. Sci..

[59] Teye M., Barku V.Y.A., Hagan J.K. (2020). Fatty acid profile, carcass and meat quality attributes of rabbit breeds in Ghana fed diets with graded levels of palm (*Elaeis guineensis*) kernel oil residue. Adv. Anim. Vet. Sci..

[60] Ebrahimi M., Rajion M.A., Goh Y.M., Sazili A.Q. (2012). Impact of different inclusion levels of oil palm (*Elaeis guineensis* Jacq.) fronds on fatty acid profiles of goat muscles. J. Anim. Physiol. Anim. Nutr..

[61] Cardoso H.M.C. (2025). Benefits of supplementation of organically complexed trace minerals (Zn, Cu, Mn and Fe) in poultry and swine: A mini-review. Res. Soc. Dev..

[62] Prado J.M., Forster-Carneiro T., Rostagno M.A., Follegatti-Romero L.A., Maugeri Filho F., Meireles M.A.A. (2014). Obtaining sugars from coconut husk, defatted grape seed, and pressed palm fiber by hydrolysis with subcritical water. J. Supercrit. Fluids.

[63] Nabi F., Arain M.A., Rajput N., Alagawany M., Soomro J., Umer M., Soomro F., Wang Z., Ye R., Liu J. (2020). Health benefits of carotenoids and potential application in poultry industry: A review. J. Anim. Physiol. Anim. Nutr..

[64] Izuddin W.I., Loh T.C., Akit H., Nayan N., Noor A.M., Foo H.L. (2022). Influence of dietary palm oils, palm kernel oil and soybean oil in laying hens on production performance, egg quality, serum biochemicals and hepatic expression of beta-carotene, retinol and alpha-tocopherol genes. Animals.

[65] Tsouko E., Alexandri M., Fernandes K.V., Freire D.M.G., Mallouchos A., Koutinas A.A. (2019). Extraction of phenolic compounds from palm oil processing residues and their application as antioxidants. Food Technol. Biotechnol..

[66] Can Z., Gidik B., Kara Y., Kolayli S. (2024). Antioxidant activity and phenolic content of bee breads from different regions of Türkiye by chemometric analysis (PCA and HCA). Eur. Food. Res. Technol..

[67] Nemli E., Günal-Köroğlu D., Apak R., Capanoglu E. (2025). Potential of plant-based oil processing wastes/by-products as an alternative source of bioactive compounds in the food industry. Foods.

[68] Mahfuz S., Shang Q., Piao X. (2021). Phenolic compounds as natural feed additives in poultry and swine diets: A review. J. Anim. Sci. Biotechnol..

[69] Abdel-Moneim E.A.M., Shehata A.M., Alzahrani S.O., Shafi M.E., Mesalam N.M., Taha A.E., Swelum A.A., Arif M., Fayyaz M., Abd El-Hack M.E. (2020). The role of polyphenols in poultry nutrition. J. Anim. Physiol. Anim. Nutr..

[70] Kotsou K., Chatzimitakos T., Athanasiadis V., Bozinou E., Lalas S.I. (2024). Exploiting agri-food waste as feed for *Tenebrio molitor* larvae rearing: A review. Foods.

[71] Bordiean A., Krzyżaniak M., Stolarski M.J. (2022). Bioconversion potential of agro-industrial byproducts by *Tenebrio molitor*—Long-term results. Insects.

[72] Harsányi E., Juhász C., Kovács E., Huzsvai L., Pintér R., Fekete G., Varga Z.I., Aleksza L., Gyuricza C. (2020). Evaluation of organic wastes as substrates for rearing *Zophobas morio*, *Tenebrio molitor*, and *Acheta domesticus* larvae as alternative feed supplements. Insects.

[73] Oonincx D.G.A.B., Van Broekhoven S., Van Huis A., Van Loon J.J.A. (2015). Feed conversion, survival and development, and composition of four insect species on diets composed of food by-products. PLoS ONE.

[74] Van Broekhoven S., Oonincx D.G.A.B., Van Huis A., Van Loon J.J.A. (2015). Growth performance and feed conversion efficiency of three edible mealworm species (Coleoptera: Tenebrionidae) on diets composed of organic by-products. J. Insect Physiol..

[75] Tamim B., Salter A., Parr T., Brameld J. (2025). Effects of feed nutrients on growth, development and the deposition of protein and fat in *Tenebrio molitor* larvae. J. Insects Food Feed.

[76] Meneguz M., Schiavone A., Gai F., Dama A., Lussiana C., Renna M., Gasco L. (2018). Effect of rearing substrate on growth performance, waste reduction efficiency and chemical composition of black soldier fly (*Hermetia illucens*) larvae. J. Sci. Food Agric..

[77] Moradei A., Spranghers T., Ottoboni M., Deruytter D., Pinotti L. (2025). Effects of four herbs on the composition and growth performance of *Tenebrio molitor* larvae. J. Insects Food Feed.

[78] Wang T., Li S., Ning J., Li J., Han Y., Yin X., Huang X., Huang F. (2023). Effects of different processing techniques of palm kernel cake on processing quality of pellet feed, nutrient digestibility, and intestinal microbiota of pigs. J. Anim. Sci..

[79] de Melo Lisboa M., Silva R.R., da Silva F.F., de Carvalho G.G.P., da Silva J.W.D., Paixão T.R., da Silva A.P.G., de Carvalho V.M., Santos L.V., da Conceição Santos M. (2021). Replacing sorghum with palm kernel cake in the diet decreased intake without altering crossbred cattle performance. Trop. Anim. Health Prod..

[80] Leong S.Y., Kutty S.R.M., Malakahmad A., Tan C.K. (2016). Feasibility study of biodiesel production using lipids of *Hermetia illucens* larvae fed with organic waste. Waste Manag..

[81] Lim J.J., Liew C.S., Raksasat R., Merican Z.M.A., Kiatkittipong K., Abdelfattah E.A., Mohamad M., Bashir M.J.K., Ntwampe S.K.O., Lim J.W. (2022). Cellulase-pretreated palm decanter cake for feeding of black soldier fly larvae in triggering bioaccumulation of protein and lipid for biodiesel production. Sustain. Energy Technol. Assess..

[82] Morales-Ramos J.Á., Rojas M.G., Kelstrup H.C., Emery V. (2020). Self-selection of agricultural by-products and food ingredients by *Tenebrio molitor* (Coleoptera: Tenebrionidae) and impact on food utilization and nutrient intake. Insects.

[83] Galassi G., Jucker C., Parma P., Lupi D., Crovetto G.M., Savoldelli S., Colombini S. (2021). Impact of agro-industrial byproducts on bioconversion, chemical composition, in vitro digestibility, and microbiota of the black soldier fly (Diptera: Stratiomyidae) larvae. J. Insect Sci..

[84] Muliari M., Zulfahmi I., Akmal Y., Karja N.W.K., Nisa C., Sumon K.A., Rahman M.M. (2020). Toxicity of palm oil mill effluent on the early life stages of Nile tilapia (*Oreochromis niloticus*, Linnaeus 1758). Environ. Sci. Pollut. Res..

[85] Bordiean A., Krzy˙zaniak M., Aljewicz M., Stolarski M.J. (2022). Influence of different diets on growth and nutritional composition of yellow mealworm. Foods.

[86] Cansee S., Suraporn S., Butwong N. (2025). Enhancing food production by sustainable cricket farming in Thailand: Evaluating black soldier fly larvae as a cost-effective feed ingredient. Insects.

[87] Seyedalmoosavi M.M., Mielenz M., Veldkamp T., Das G., Metges C.C. (2022). Growth efficiency, intestinal biology, and nutrient utilization and requirements of black soldier fly (*Hermetia illucens*) larvae compared to monogastric livestock species: A review. J. Anim. Sci. Biotechnol..

[88] Yuan Y., Li L., Zhao J., Chen M. (2020). Effect of tannic acid on nutrition and activities of detoxification enzymes and acetylcholinesterase of the fall webworm (Lepidoptera: Arctiidae). J. Insect Sci..

[89] Leni G., Maistrello L., Pinotti G., Sforza S., Caligiani A. (2023). Production of carotenoid-rich *Hermetia illucens* larvae using specific agri-food by-products. J. Insects Food Feed..

[90] Malla N., Nørgaard J.V., Roos N. (2023). Protein quality of edible insects in the view of current assessment methods. Anim. Front..

[91] Pittarate S., Arjin C., Vivekanandhan P., Swathy K., Chiu C.I., Mekchay S., Krutmuang P. (2025). Evaluating the nutrient and fatty acid profiles of black soldier fly larvae (*Hermetia illucens*) raised on various diets in Thailand. Front. Insect Sci..

